# Editorial

**Published:** 1951-10

**Authors:** 


### Editorial

By the time these pages reach our readers the direction of the *Journal* will have passed into other hands. The Committee of the Society has on this occasion gone back to the principles that directed the first appointment. Mr. H. K. Lucas is a newcomer to Bristol, to the Society, and to clinical journalism. His ideas and ideals have not had time to lose their freshness during a long apprenticeship. We feel sure that all our readers will join in congratulating him on his appointment and themselves on securing his services.

